# T cell pathology in skin inflammation

**DOI:** 10.1007/s00281-019-00742-7

**Published:** 2019-04-26

**Authors:** Robert Sabat, Kerstin Wolk, Lucie Loyal, Wolf-Dietrich Döcke, Kamran Ghoreschi

**Affiliations:** 1 Psoriasis Research and Treatment Center, Department of Dermatology, Venereology and Allergology/Institute of Medical Immunology, Charité – Universitätsmedizin Berlin, Charitéplatz 1, 10117 Berlin, Germany; 2 Berlin-Brandenburg Center for Regenerative Therapies (BCRT), Charité – Universitätsmedizin Berlin, Augustenburger Platz 1, 13353 Berlin, Germany; 3 SBU Oncology, Pharmaceuticals, Bayer AG, Berlin and Wuppertal, Müllerstraße 178, 13353 Berlin, Germany; 4 Department of Dermatology, Venereology and Allergology, Charité – Universitätsmedizin Berlin, Charitéplatz 1, 10117 Berlin, Germany

**Keywords:** Skin inflammation, Immune-mediated disease, IL-4, IL-17, IL-22, IFN-γ, TNF-α, TGF-β

## Abstract

Forming the outer body barrier, our skin is permanently exposed to pathogens and environmental hazards. Therefore, skin diseases are among the most common disorders. In many of them, the immune system plays a crucial pathogenetic role. For didactic and therapeutic reasons, classification of such immune-mediated skin diseases according to the underlying dominant immune mechanism rather than to their clinical manifestation appears to be reasonable. Immune-mediated skin diseases may be mediated mainly by T cells, by the humoral immune system, or by uncontrolled unspecific inflammation. According to the involved T cell subpopulation, T cell–mediated diseases may be further subdivided into T1 cell–dominated (e.g., vitiligo), T2 cell–dominated (e.g., acute atopic dermatitis), T17/T22 cell–dominated (e.g., psoriasis), and Treg cell–dominated (e.g., melanoma) responses. Moreover, T cell–dependent and -independent responses may occur simultaneously in selected diseases (e.g., hidradenitis suppurativa). The effector mechanisms of the respective T cell subpopulations determine the molecular changes in the local tissue cells, leading to specific microscopic and macroscopic skin alterations. In this article, we show how the increasing knowledge of the T cell biology has been comprehensively translated into the pathogenetic understanding of respective model skin diseases and, based thereon, has revolutionized their daily clinical management.

## Introduction

As the barrier between the organism and the environment, the skin protects against external hazards, noxious substances, and pathogens. It is the largest and heaviest human organ and is built up by three distinct layers: the outermost epidermis, the dermis, and the subcutis [1–3]. The numerically dominant cell population of the continuously renewing epidermis is that of keratinocytes. Proliferation of these cells usually takes place within the *stratum basale* (attached to the basement membrane). Here, the epidermal stem cells are situated, which, upon their rare divisions, deliver the so-called transit amplifying cells (TA cells), a frequently proliferating population [1, 4]. Each division of an individual TA cell delivers a daughter cell able to leave the basement membrane and to start terminal differentiation in the suprabasal *stratum spinosum*. During terminal differentiation, these cells move upward, becoming granular (*stratum granulosum*) and undergoing a particular apoptotic process to become horny constructs. Tightly covering the skin surface, these so-called corneocytes form the uppermost layer of the epidermis, the *stratum corneum* [1–3]. Under normal conditions, there is a balance between stem cell proliferation, TA cells, terminal differentiation, and the continuous desquamation of corneocytes from the skin surface (about 50 billion daily). This equilibrium is markedly disrupted in some chronic immune-mediated skin diseases [5]. Besides keratinocytes, Merkel cells, melanocytes, and immune cells, including Langerhans cells and resident memory CD8+ T cells, are also present in the epidermis [6, 7]. The dermis, lying under the epidermis, consists of connective tissue containing collagenous, elastic, and reticular fibers as well as fibroblasts and hosts immune cells like macrophages, immature dendritic cells (DCs), mast cells, and some resident memory CD4+ T cells.

The permanent contact of the skin with exogenous stimuli and antigens frequently leads to activation of the resident immune cells. The cutaneous persistence of the stimulus/antigen and/or a relative deficiency of counter-regulatory mechanisms, particularly in the context of a genetic predisposition, results in local immune cell infiltration and chronic activation, which also involves the cutaneous tissue cells. Hence, it is not surprising that chronic immune-mediated skin diseases are some of the most common disorders in humans. For the affected patients, these diseases induce not only physical but also psychological burdens due to the visibility of the symptoms and the frequent association with itching, pain, and burning [8–10]. They may be primarily mediated by the uncontrolled activation of T cells, the humoral immune system, or unspecific inflammation (innate immunity). Disorders dominated by pathogenic CD4+ and/or CD8+ T cells comprise the largest group within the chronic immune-mediated skin diseases [11]. A deeper understanding of the molecular and cellular mechanisms underlying these disorders might lead to the identification of novel target molecules and, as a consequence, to the development of innovative therapeutic strategies. In this review, we will discuss the mechanisms of development and maintenance of specialized T cell subtypes and refer to representative diseases, in which the specific T cell subtypes play a crucial pathogenic role.

## Characteristics, development, and functions of T cell subpopulations

T cells, a central component of the adaptive immunity, play a pivotal role in the defense against pathogens and tumors, while their dysregulation contributes to the development and maintenance of various diseases. T cells mature in the thymus, where they undergo somatic gene rearrangement resulting in the expression of a unique T cell receptor (TCR) [12]. During the positive selection process, detection of antigens presented on major histocompatibility complex class 1 (MHCI) or class 2 (MHCII) by the rearranged TCR implements either a CD8+ or CD4+ T cell lineage fate, respectively [13]. Presentation of autoantigens in the thymic medulla ensures the elimination of autoreactive T cells [14], and remaining T cells egress into circulation where they patrol blood and lymph as CD45RA+CCR7+ naïve T cells [15].

When T cells bind their cognate antigen by the TCR accompanied by a sufficient co-stimulatory signal, they become activated, start proliferating, and contribute to pathogen clearance as effector cells [16]. After pathogen clearance, 95% of the effector cells undergo apoptosis; the remaining T cells give rise to a highly specialized set of memory cells that have lost CD45RA expression and instead express CD45RO [17]. The memory compartment can be subdivided into CCR7+ central memory (T_CM_), CCR7- effector memory (T_EM_), and CCR7- effector memory T cells re-expressing CD45RA (T_EMRA_) [15]. While T_CM_ migrate through lymphatic tissue and were described to be less responsive, T_EM_ patrol peripheral tissues and provide rapid effector function upon reactivation [15]. Single-cell-based experiments suggest a progressive maturation of T cells from naive via T_CM_ and T_EM_ to T_EMRA_ cells that are associated with chronic activation and display features of exhaustion such as impaired cytokine secretion and the expression of exhaustion markers such as programmed death-1 (PD-1) and TIM3 [18–20]. Upon migration into the different lymphoid and non-lymphoid tissues, some memory T cells gain tissue residency characterized by the upregulation of CD69 and CD103, the hallmarks of tissue-resident memory T cells (T_RM_), as well as expression of Hobit and Blimp1, which together suppress the re-egress into circulation [21]. Those cells exhibit an outstanding long-term maintenance and act as sentinels that protect against re-infections.

Antigen detection by CD4+ T cells is restricted to MHCII expressed on professional antigen-presenting cells (APCs) including dendritic cells (DCs), B cells, and macrophages [22]. These APCs continuously sample proteins and present peptides derived thereof on their surface. In the presence of conserved pathogenic structures or danger signals that are released by distressed cells, they become activated and migrate to the secondary lymphoid organs, where they in turn activate CD4+ T cells [23, 24]. A central role of CD4+ T cells is to migrate into B cell follicles upon activation, where they mediate B cell help by CD40L:CD40 interaction. This CD40L-mediated help is indispensable for the induction of germinal center formation, antibody class switch, and somatic hypermutation [25]. During T cell activation, the cytokine milieu at the site of infection moreover modulates the differentiation and subsequent specialization of the T cells, allowing pathogen-tailored responses. The cytokines IL-12 and IFN-γ induce the expression of the transcription factor T-bet in T cells, resulting into IFN-γ-producing type 1 helper (Th1) T cells that contribute to the clearance of virus-infected cells and intracellular pathogens [26]. Induction of Gata3 expression by IL-4 gives rise to type 2 helper (Th2) cells secreting IL-4, IL-5, and IL-13, which are critical mediators of extracellular parasite expulsion and mediate B cell class switch [27]. In recent years, the spectrum of CD4+ T cell subsets rapidly broadened by the identification of type 17 and type 22 helper (Th17, Th22), T follicular helper (Tfh) and regulatory (Treg) T cells. Th17 cells differentiate upon RORγt expression and produce IL-17 that induces epithelial antimicrobial defense and leads to recruitment and activation of neutrophils [28]. The work of Acosta-Rodriguez et al. suggests that IL-17-producing cells are a heterogeneous population consisting of Th17 cells dominating anti-fungal responses, whereas Th17+1 cells additionally secrete IFN-γ and are the main responders in the defense of extracellular bacteria [29]. Th22 cells differentiate upon aryl hydrocarbon receptor expression, act on epithelial cells like keratinocytes by IL-22 secretion, and promote wound healing and tissue protection against damage [30]. Upon activation, some naïve CD4+ T cells upregulate the transcription factor Bcl6 and migrate into B cell follicles where they become resident Tfh cells contributing to germinal center formation [31, 32]. In contrast, FoxP3+ Treg cells do not contribute to pathogen defense but instead prevent autoimmune disorders by suppressing unwanted immune responses [33]. The major population of CD4+ Treg cells was found to be characterized by high expression of the IL-2 receptor alpha chain (CD25), and the transcription factor FoxP3, the latter being indispensable for the development and suppressive function of Treg cells [34, 35].

Extensive analyses of the CD4+ T cell subsets revealed differing migration abilities, which are reflected by the expression of unique sets of chemokine receptors that mediate migration along a chemokine gradient. Combinations of the chemokine receptors CCR4, CCR6, CCR10, and CXCR3 were identified as separators of Th1 (CCR6-CCR4-CXCR3+), Th2 (CCR6-CCR4+CXCR3-), Th17+1 (CCR6+CCR4-CXCR3+), Th17 (CCR6+CCR4+CXCR3-CCR10-), and Th22 (CCR6+CCR4+CXCR3-CCR10+) cells [29, 36, 37]. CCR6 expression is—together with CD161—a common feature of IL-17-secreting cells [29, 38, 39]. The ligand for CCR6 is CCL20, which is predominantly produced by epithelial cells, organ-associated lymphoid tissues, and liver, allowing a broad migration pattern that is specified by the co-expression of further chemokine receptors [40]. In contrast, CCR4 and CCR10 expression is implemented by DCs in skin-draining lymph nodes and allows the chemotactic migration along CCL17/CCL22 and CCL27/28, respectively [41, 42]. CCR4- and CCR10-expressing T cells co-express the so-called cutaneous leukocyte-antigen (CLA) and altogether mediate homing into the skin [43]. CXCR3 binds to CXCL9, CXCL10, and CXCL11, which are secreted in the presence of IFN-γ and recruit CXCR3+ cells to sites of inflammation (reviewed in [44]). Beyond, the expression of CXCR5 is characteristic of Tfh cells, which binds the chemokine CXCL13 secreted by the follicular stroma, allowing the recruitment into the B cell follicle zones [45]. This concerted differentiation of T cells orchestrated by DCs ensures the right response at the right place in the body. CD4+ T cells possess a broad flexibility regarding the subset they differentiate into. By comparison of the TCR clone repertoire in *Mycobacterium tuberculosis* and *Candida albicans* infection, Becattini et al. could demonstrate overlaps in the clones found in the different CD4+ helper subsets, suggesting that priming of a single naïve CD4+ T cell can give rise to multiple fates [46].

**Fig. 1:**
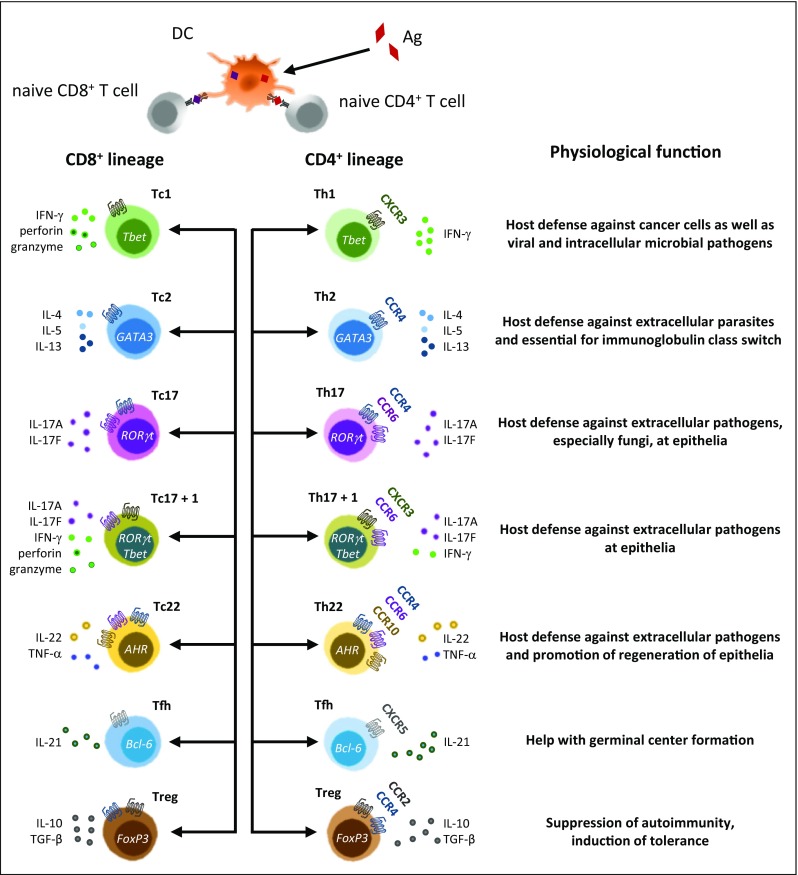
Phenotype and function of T cell subsets

In contrast to CD4+ T cells, CD8+ T cells were described cytotoxic T lymphocytes (Tc) that directly kill malign or infected cells. They detect antigens presented by MHCI, which is expressed by almost every cell in the body, to either eliminate the cell by the secretion of cytolytic molecules including perforin and granzymes or to induce Fas-mediated apoptosis (reviewed in [47]). In the memory stage, most (Tc1) cytotoxic CD8+ T cells express the transcription factor T-bet and secrete high levels of IFN-γ. However, some CD8+ T cells were identified that express Gata3 and display a type 2 cytotoxic (Tc2) T cell phenotype with secretion of IL-4, IL-5, and IL-13. Comparable with their CD4+ Th2 counterparts, they possess a CCR4+ and CRTH2+ phenotype [48]. Effector profiles of CD8+ T cells in multiple diseases such as *psoriasis vulgaris* demonstrated that, among memory CD8+ T cells, also IL-17, IL-22, and IL-17/IFN-γ producers exist [49]. We could demonstrate that the CD8+ T cell subsets Tc1, Tc2, Tc17, Tc1+1, and Tc22 express the same set of chemokine receptors and utilize the same differentiation programs based on T-bet, Gata3, RORγt, and aryl hydrocarbon receptor as do CD4+ T cells (Loyal et al., manuscript under review). While Tc1 and Tc17+1 CD8+ T cells display a classical cytotoxic phenotype, Tc2, Tc17, and Tc22 lack the capability to kill target cells and express the Th cell–typical molecule CD40L instead (Loyal et al., manuscript under review; [50]). In contrast to CD4+ T cells, the differentiation flexibility is restricted among CD8+ T cells, with a certain flexibility to gain Tc1 or Tc17+1 phenotype on the one side or to gain Tc2, Tc17, or Tc22 phenotype on the other side, but with very little clonal overlap between these two groups (Loyal et al., manuscript under review). They share the ability to secrete IL-13 and provide CD40L-dependent help (Loyal et al., manuscript under review; [50]). This striking effect might be caused by the site of priming, the involved APC, the priming conditions, and especially the type of antigen that gives rise to non-cytotoxic, “helper-type” CD8+ T cells. Their chemokine receptor expression, effector profile, and lack of cytotoxicity suggest a tissue homeostasis–maintaining function instead of contribution to the elimination of infected/malign/distorted cells. Cheuk et al. demonstrated that, in human skin, a significant fraction of CD8+ T cells lack cytotoxic features including the expression of CD49a and instead produce IL-17 [51]. In a murine model, those skin Tc17 cells were shown to contribute to wound healing by IL-13 release upon recognition of non-classical MHCI (H2-M3)-presented peptides derived from commensal bacteria [52, 53]. Altogether, CD4+ and CD8+ T cells provide a broad repertoire of highly specific features and functions adapted to the diverse spectrum of challenges such as infections but also tissue homeostasis, wound healing, and tolerance as summarized in Fig. 1.

Besides their role in pathogen defense, the activation of skin-directed T cells can lead to chronic T cell–mediated skin diseases, whose characteristics correspond to the specific effector mechanisms of the different T cell subpopulations. In fact, those diseases may be dominated by a Th/c1-specific (e.g., vitiligo), Th/c2-specific (e.g., acute stage of atopic dermatitis), or Th/c17-/Th/c22-specific (e.g., psoriasis) pattern. Moreover, the activation of Treg cells is associated with skin tumors like melanoma, a malignant condition derived from melanocytes. Finally, T cell responses may be paralled by non-T cell responses, as in the case of hidradenitis suppurativa, where T17 cell activation [54, 55], a relative deficiency in Treg cells [56], and strong unspecific inflammation [57] have been found.

Since, as described above, the classical division of T cells into CD4+ helper T cells and CD8+ cytotoxic T cells has become out of date, the following sections will use the terms T1, T2, T17, T22, and Treg cells. The role of specialized T cells in representative skin diseases will be discussed in the following.

## Common T cell–related skin disorders

### T1 cells and vitiligo

#### Epidemiology, clinical, and histological characteristics

Vitiligo is a chronic disease characterized by the appearance of pigment-free patches of the skin and rarely of the mucosa. In Europe, about 0.5–2% of people suffer from vitiligo. However, the prevalence of vitiligo in, e.g., India and Arabic countries, is appreciably higher [58, 59]. Both sexes are equally affected, and a quarter of the cases concern children [60]. Typically, the vitiligo patches are sharply demarcated and differ in shape and size [58, 61]. However, apart from the depigmentation, they are macroscopically very similar to intact skin. Periorbital, perioral, and acral regions of the body are often affected. During the course of the disease, the number and size of the depigmented patches can increase, and patches can coalesce. As expected from the loss of pigment, the histological examination of vitiligo patches shows lack of melanocytes and melanin-containing keratinocytes in the *stratum basale* [61, 62]. During the early stage, a perivascular lymphocyte infiltration is observed in the dermis. In the late stage, though, the lymphocytes are mainly present at the edge of the patches [62].

#### Immunopathophysiology of vitiligo

Every tenth cell in the *stratum basale* of healthy skin is a melanocyte, and - under physiological conditions - melanocytes are not attacked by the immune system. However, melanocytes can be targeted or even destroyed by T cell–mediated immune responses initiated by autoimmune processes or therapeutic intervention. Like in other autoimmune diseases, genetic predisposition is also present in individuals with vitiligo. Besides certain HLA genotypes, patients with vitiligo can show single nucleotide polymorphisms (SNPs) within genes that are implicated in T cell signaling or activation (*NLRP1*, *TICAM1*, *FOXP3*, *BACH2*, *PTPN22*, *CD80*) or genes associated with cytotoxic T cell responses (*GZMB*, *IL2RA*) [60]. Nonetheless, the role of genetic predisposition in vitiligo etiology seems to be less important than that in other chronic T cell–mediated diseases like psoriasis or atopic dermatitis. In fact, only 1–10% of vitiligo patients have a positive family history for vitiligo in contrast to about 30% in the case of psoriasis.

It is generally accepted that the first step in vitiligo pathogenesis is a slight damage of melanocytes, e.g., by ultraviolet (UV) radiation or chemical substances. Such damage leads to an increase of reactive oxygen species (ROS), in particular when low levels of enzymatic and non-enzymatic antioxidants are present [63]. In fact, the impairment of the nuclear factor E2-related factor 2 (Nrf2), a protein important for protection against oxidative stress, seems to be critical for the increased sensitivity of vitiligo melanocytes to oxidative stress [64] as observed in lesional and non-lesional skin of patients. ROS and respective chemical substances provoke alteration of the folding machinery of the endoplasmic reticulum, leading to accumulation of immature proteins and finally to autophagy or apoptosis [60]. The increase of ROS is associated with the release of melanocyte-specific antigens and molecules like heat-shock proteins (HSPs) and self RNA/DNA, which activate pathogen recognition receptors on macrophages and DCs [63]. As reported, inducible HSP70 promotes an inflammatory DC phenotype and accelerates disease progression in a murine model of vitiligo [65].

**Fig. 2:**
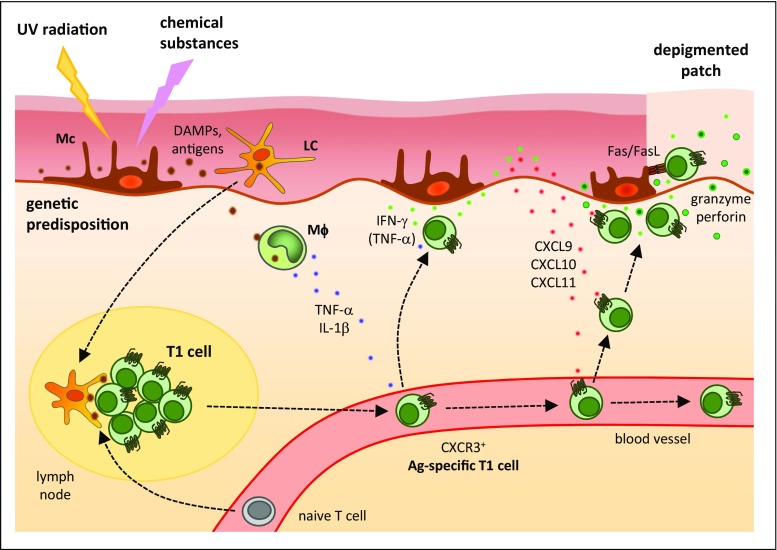
Immunopathophysiology of vitiligo

The described events induce generation of T1 cells in lymph nodes that are specific for melanocyte antigens (Fig. 2). The infiltration of such T cells into the skin seems to depend on the chemokine receptor CXCR3 expressed by T1 cells and its ligands CXCL9, CXCL10, and CXCL11 produced by cutaneous tissue cells like keratinocytes [66]. Interestingly, vitiligo mouse models suggest that CXCL9 promotes Tc1 recruitment into the skin but not their effector function, whereas CXCL10 is required for effector function [67]. In the progressive phase of the disease, the immigrated T1 cells, in particular Tc1 cells, destroy melanocytes through the production of IFN-γ and TNF-α as well as cytotoxic molecules like granzyme B and perforin [51] (Fig. 2). In fact, Tc1 cells isolated from the edges of patches induced apoptosis in autologous melanocytes in co-cultures in vitro [68]. Furthermore, IFN-γ induces CXCL9 and CXCL10 in cutaneous tissue cells [69]. In contrast, the Treg cell response in the skin of patients with vitiligo seems to be limited [70], so that Treg cells are not able to prevent the cytotoxic IFN-γ-dominated T1 cell response [71]. Of note, individuals with vitiligo have a lower risk for developing malignant melanoma (see below). This observation shows that immune activation directed against melanocytic antigens can be of benefit in the setting of carcinogenesis.

Besides IFN-γ, IL-17 expression is also increased in perilesional skin of vitiligo patients, where T cells appear as the main source of this cytokine [72]. Since depigmentation is not a typical finding in psoriasis, a disease with high IL-17 expression in the skin, it is questionable, whether IL-17 significantly contributes to vitiligo pathogenesis.

#### Immunopathology-based therapy of vitiligo

Treating vitiligo is a challenge, since no systemic therapies are yet available. Understanding the exact pathogenetic processes in vitiligo could help in developing successful therapeutic strategies (Table 1). The dominant role of IFN-γ in the depigmentation in mouse models of vitiligo [73] suggests that neutralizing this cytokine, inhibiting its production or signaling pathway, may help to stop the disease. More recently, two case reports described that patches rapidly repigmented in vitiligo patients treated with JAK inhibitors like tofacitinib or ruxolitinib that interfere with IFN-γ signaling [74]. Since cumulating reports show that pathogenic T1 cells in vitiligo are tissue-resident memory T cells, interventions focused on IFN-γ neutralization or hindrance of the effect of this cytokine should be periodically repeated [51]. Besides IFN-γ as cytokine factor, T cell–based cytotoxic mechanisms are involved in melanocyte destruction. Thus, the depletion of T1 cells or the inhibition of their migration into the skin may result in promising approaches. The minimization of skin infiltration by T1 cells might be achieved by inhibiting CXCR3 function, as demonstrated in experimental mice [67]. Targeting the CXCR3 chemokine receptor to deplete T1 cells from skin is another alternative approach, as also recently demonstrated in mice [75]. Interestingly, this latter approach did not only prevent depigmentation but also lead to perifollicular re-pigmentation.

**Table 1:** Pipeline of drugs for systemic treatment of vitiligo

Target(s)	Drug name	Phase	Company	Trial ID	Study start	Status
CD80/CD86	Abatacept	1	Bristol-Myers Squibb	NCT02281058	1.2015	Active, not recruiting
PDE4	Apremilast	2	Celgene	NCT03036995	3.2017	Active, not recruiting
Jak3	PF-06651600	2b	Pfizer	NCT03715829	11.2018	Recruiting
Tyk2/Jak1	PF-06700841	2b	Pfizer	NCT03715829	11.2018	Recruiting

Source: Clinicaltrials.gov. Clinical trials that started after January 2012 are shown

### T2 cells and atopic dermatitis

#### Clinical and histological picture of atopic dermatitis

A more frequent T cell–mediated skin disease than vitiligo is atopic dermatitis. It usually begins in infancy. Its prevalence is very high in the Western population, with 15–20% of children and 3–4% of adults being affected [10]. The clinical manifestation of atopic dermatitis is age- and stage-dependent. While, in infants, skin lesions occur especially in the face and on the scalp, at later age, the flexural surfaces of the elbows and knees, the hands, feet, and the neck are increasingly affected. Acute lesions present as strongly itchy with red papules, serous exudation, and crusting. Histologically, edemas, vesiculation, and moderate hypogranularity and hyperkeratosis can be observed in the epidermis. Immune infiltration of the skin includes T cells, mast cells and eosinophilic granulocytes, macrophages, and DCs. Chronic lesions show increased collagen deposition in the dermis resulting in skin lichenification. Microscopically, acanthosis and more macrophage-dominated dermal infiltrations are visible at this stage. In contrast to psoriasis, lesions are less clearly demarcated [76]. In addition to the cutaneous alterations, 80% of patients suffer from allergies and often develop allergic asthma and rhinitis (extrinsic disease) [77].

#### Immunopathophysiology of atopic dermatitis

**Fig. 3:**
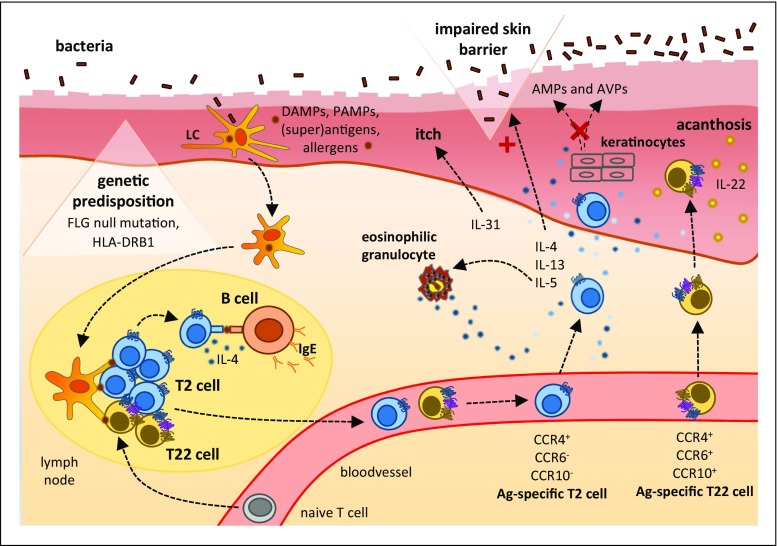
Immunopathophysiology of atopic dermatitis

Atopic dermatitis has a multifactorial nature with a genetic component and environmental factors being involved (Fig. 3). A positive family history has been reported in 40–60% of patients [78, 79]. The strongest genetic association concerns the gene encoding the skin-barrier molecule filaggrin (*FLG*). In fact, 20–30% of patients carry a *FLG* null mutation [80]. This matches the fact that the impaired skin barrier is an essential factor in the pathogenesis and correlates with the severity of this disease [81]. Atopic dermatitis has also been linked to variants within the genes encoding the T2 pathway-associated cytokines/cytokine receptors IL-4, IL-13, IL-4RA, and IL-31 and associated downstream molecules like STAT6 and GATA3 [82]. A characteristic MHC variant reported in some patient populations with atopic dermatitis is HLA-DRB1 [83]. Exogenous triggers of the disease include allergens, microbial antigens/superantigens, mental stress, and scratching of the skin [76].

T2 cell mediators are crucial for the pathogenesis of atopic dermatitis [80] (Fig. 3). At the chronic disease stage, the T22 mediator IL-22 is also of relevance [84, 85]. IL-4, IL-13, IL-31, and IL-22 seem to interfere with keratinocyte terminal differentiation [86–89]. This may explain the decreased epidermal expression of filaggrin and other molecules necessary for skin differentiation and barrier function even in patients without *FLG* mutation. IL-31 is involved in the pathophysiology of itching, a characteristic finding patients with atopic dermatitis suffer from. IL-5 is known to activate eosinophilic granulocytes that are found histologically in skin lesions from the patients. IL-4 also promotes production of IgE, which shows elevated serum levels in the majority of patients with atopic dermatitis [80]. Chronic lesions also express moderate levels of the T1 cell cytokine IFN-γ, while IL-17 can hardly be detected [76, 90].

The aforementioned cytokine pattern is also the reason for the deficient epidermal production of anti-microbial proteins (AMPs) and antiviral proteins (AVPs) atopic dermatitis patients show (Fig. 3). In fact, high T2 cytokine/low IL-17 levels result in low AMP production by keratinocytes [91–93], while lacking expression of the T17 cytokine IL-29 is associated with impaired keratinocyte AVP expression [94] (see also “Common T cell–related disorder”). Low AMP levels in the barrier-disturbed skin of atopic dermatitis patients predestinate for atypical cutaneous colonization with *Staphylococcus aureus*, penetration of microbial pathogens and their immunostimulating constituents into the skin, and infections with this pathogen [95–99]. Interestingly, subclinical *S. aureus* colonization also occurs in non-lesional skin of patients, correlating with disturbed skin barrier function and disease extent [99, 100]. Atopic dermatitis patients also show an increased risk of developing skin infections with viral pathogens, including human papillomavirus, herpes simplex virus (HSV), and *molluscum contagiosum* virus [95, 98]. In rare cases, HSV infection may spread and cause *Ekzema herpeticatum* [101]. Importantly, the impaired skin barrier function also promotes epicutaneous sensitization to allergens and may explain the high allergy frequency in affected patients.

#### Immunopathology-based therapy of atopic dermatitis

Classically, topical corticosteroids and, for more severe disease, systemic immunosuppressive agents are used. Since 2016, topical phosphodiesterase inhibitors such as crisaborole [102] (for the treatment of mild to moderate disease) are approved. More recently, the first biologic for treating atopic dermatitis, namely dupilumab, was introduced [103]. This antibody targets the IL-4/IL-13 receptor and was approved in 2017 for the treatment of moderate to severe disease (Table 2). A large number of other biologics for the treatment of atopic dermatitis are under development (Table 3).

**Table 2:** Approved biologics for the treatment of atopic dermatitis, psoriasis, and melanoma

Indication	Target	Drug name
AD	IL-4Rα	Dupilumab
Pso, PsA	TNF-α	Etanercept
Pso, PsA	TNF-α	Infliximab
Pso, PsA	TNF-α	Adalimumab
PsA	TNF-α	Golimumab
Pso, PsA	TNF-α	Certolizumab-pegol
PsA	CD80/CD86	Abatacept
Pso, PsA	p40	Ustekinumab
Pso, PsA	IL-17	Secukinumab
Pso, PsA	IL-17	Ixekizumab
Pso	IL-17R	Brodalumab
Pso	p19	Guselkumab
Pso	p19	Tildrakizumab
Melanoma	CTLA-4	Ipilimumab
Melanoma	PD-1	Pembrolizumab
Melanoma	PD-1	Nivolumab
Melanoma	IFN-αR	IFN-α-2b
Melanoma	IL-2R	IL-2

AD *atopic dermatitis*, *Pso* psoriasis, *PsA* psoriasis arthritis

**Table 3:** Biologics under development for the treatment of atopic dermatitis

Target(s)	Drug name	Phase	Company	Trial ID	Study start	Status
IgE	Ligelizumab	2	Novartis	NCT01552629	1.2012	Completed
IgE	Anti-CemX	2	Fountain BioPharma	NCT03758716	11.2018	Active, not recruiting
IgE, FcγRIIb	XmAb7195	1	Xencor	NCT02148744	9.2015	Completed
IL-1α	Bermekimab	2	XBiotech	NCT03496974	11.2018	Recruiting
IL-5	Mepolizumab	1/2	GlaxoSmithKline	NCT03055195	3.2017	Terminated
IL-5RA	Benralizumab	2	AstraZeneca	NCT03563066	9.2018	Not yet recruiting
IL-12/IL-23 (p40)	Ustekinumab	2	Janssen Pharmaceutical K.K.	NCT01945086	9.2013	Completed
IL-13	Tralokinumab	3	LEO Pharma	NCT03526861	6.2018	Recruiting
IL-13	Lebrikizumab	2	Dermira	NCT03443024	1.2018	Active, not recruiting
IL-17A	Secukinumab	2	Novartis	NCT02594098	11.2015	Completed
IL-17C	MOR106	2	Galapagos NV	NCT03568071	4.2018	Recruiting
IL-22	Fezakinumab	2	Pfizer	NCT01941537	10.2013	Active, not recruiting
IL-22R	ARGX-112	1	LEO Pharma	NCT03514511	5.2018	Recruiting
IL-23	Risankizumab	2	Abbvie	NCT03706040	12.2018	Recruiting
IL-31	BMS-981164	1	Bristol-Myers Squibb	NCT01614756	7.2012	Completed
IL-31RA	Nemolizumab	2	Galderma	NCT03100344	6.2017	Completed
IL-33	REGN3500	2b	Sanofi/Regeneron Pharmaceuticals	NCT03738423	11.2018	Recruiting
IL-33	Etokimab	2	AnaptysBio, Inc.	NCT03533751	5.2018	Recruiting
OX40	KHK4083	2	Kyowa Hakko Kirin Pharmaceutical Development, Inc.	NCT03703102	10.2018	Recruiting
OX40	GBR 830	2	Glenmark Pharmaceuticals	NCT03568162	5.2018	Recruiting
OX40	KY1005	2	Kymab Limited	NCT03754309	12.2018	Recruiting
ST2	MSTT1041A	2	Roche	NCT03747575	1.2019	Recruiting
ST2	CNTO 7160	1	Janssen Research & Development, LLC	NCT02345928	8.2014	Completed
TSLP	Tezepelumab	2b	AstraZeneca	NCT03809663	1.2019	Not yet recruiting
TSLPR	MK-8226	1	Merck Sharp & Dohme Corp.	NCT01732510	12.2012	Terminated (business reasons)
Undisclosed target	REGN846	1/2	Sanofi/Regeneron Pharmaceuticals	NCT01605708	6.2012	Terminated
Undisclosed target	LY3454738	1	Eli Lilly and Company	NCT03750643	11.2018	Recruiting

Source: Clinicaltrials.gov. Clinical trials that started after January 2012 are shown

### T17 cells, T22 cells, and psoriasis

#### Clinical and histological picture of psoriasis

With a prevalence of 2–3% in Western countries, psoriasis is another very common T cell–mediated skin disease [104]. Psoriasis manifests with sharply demarcated, raised, erythematous plaques covered by silvery scales. Lesions preferentially develop in mechanically stressed areas such as the extensor sides of the arms and legs, the sacral region, and the head [105].

Microscopically, psoriatic skin lesions show a massively thickened epidermis. This is the result of a substantial elongation of the epidermal rete ridges and an increased *stratum corneum* (hyperkeratosis). Furthermore, a reduced *stratum granulosum* and presence of nuclear remnants in the *stratum corneum* (parakeratosis) are typical features. Mechanistically, these changes are based on excessive proliferation of basal keratinocytes (TA cells) and an impaired cornification process of the keratinocytes of the upper epidermal layers [106]. In the dermis, dilatated blood capillaries greatly extend between epidermal rete ridges toward the skin surface [107]. The massive immune cell infiltration, which is most prominent in the dermis but not restricted to it, predominantly consists of monocytes/macrophages, dendritic cells, and T cells [108]. There are also accumulations of partially netose-forming neutrophilic granulocytes in the *stratum corneum*, called Munro’s microabscesses [109].

Interestingly, inflammation in psoriasis is not restricted to the skin. More than 20% of patients show involvement of the joints [110]. In addition, the prevalence of colitis is increased, and metabolic and cardiovascular alterations lead to a shortened life expectancy in the patients [111, 112].

#### Immunopathophysiology of psoriasis

**Fig. 4:**
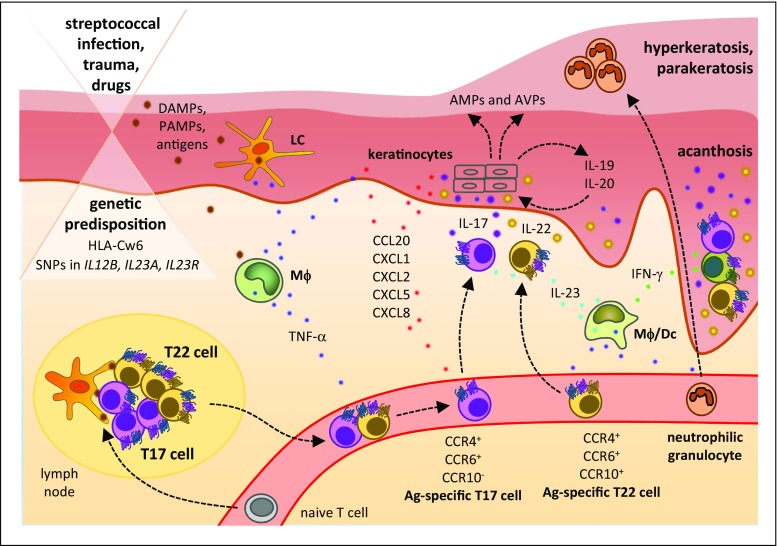
Immunopathophysiology of psoriasis

Both genetic and extern/lifestyle factors are involved in the development of psoriatic skin alterations (Fig. 4). Approximately 75% percent of patients report a positive family history [113]. A great proportion of patients carry the MHC haplotype HLA-Cw6 [113, 114], which has been correlated with certain clinical characteristics and therapeutical outcome in patients with psoriasis [115]. In addition, proposed autoantigens in psoriasis like the cathelicidin-derived peptide LL-37 and the melanocytic protein ADAMTSL5 were demonstrated to have T cell–stimulatory activity in HLA-Cw6-carrying patients [115]. Furthermore, there are associations with genes related to the keratinocyte terminal differentiation, antimicrobial defense, and the T17 cell pathway [116]. Regarding the latter (see also below), psoriasis has been linked for example to polymorphisms within *IL12B*, *IL23A*, *IL23R*, and, in some patients with a special psoriasis subtype, pustular psoriasis, also *IL36RN*. Moreover, there are associations with variants in *REL*, *TYK2*, *RUNX3*, *STAT3*, and *TRAF3IP2* [115]. Exogenous triggering factor for psoriasis involves mechanical skin trauma, streptococcal infections, and certain drugs [115].

The central pathways crucial to psoriasis pathogenesis involve T17 and T22 cells, whose mediators and upstream and downstream molecules are highly present in the lesions (Fig. 4). In addition to T cells (CD4+ and CD8+), type 3 innate lymphoid cells  play a role as producers of IL-17 and IL-22 [117, 118]. One of the most relevant cytokines promoting IL-17 and IL-22 expression by immune cells is IL-23. This heterodimeric cytokine is highly expressed in psoriatic skin. IL-23 inhibits IL-10 production by T17 cells and instead induces an inflammatory T17 phenotype [119]. Moreover, TNF-α, primarily secreted by T17 cells, T22 cells, and macrophages, as well as the T1 cell mediator IFN-γ are abundant in the psoriatic skin [120]. In sharp contrast, IL-4 is not found in psoriatic lesions.

Main target cells of IL-17A, IL-17F, and IL-22 in the skin are keratinocytes, although IL-17 effects were also described for immune cells and other tissue cells. In keratinocytes, IL-17 induces the production of selected chemokines (such as CCL20, which attracts T17 cells, T22 cells, and DCs, as well as CXCL1, CXCL2, CXCL5, and CXCL8, which all attract neutrophilic granulocytes) and other cytokines (such as IL-6, the granulocyte-activating cytokine G-CSF, and IL-19) in the skin. Presumably, IL-17 alone causes only moderate cellular responses while mainly synergizing with TNF-α, and IL-22 [121–127]. Together with IL-22, IL-17 induces the production of AMPs and therefore plays an essential role in the remarkable immune defense of the psoriatic plaque against extracellular bacteria and fungi [54, 126, 128–130]. In fact, it is a peculiarity of psoriasis patients that the impairment of the skin barrier function is not associated with an increased skin infection risk [95, 96]. Apart from its function in antibacterial defense, IL-22 is the main mediator of the impaired keratinocyte cornification process in psoriasis. IL-22 reduces the expression of molecules like filaggrin required for the terminal differentiation of keratinocytes [89]. The consequences of the IL-22-mediated inhibition of the keratinocyte terminal differentiation are reflected by a marked epidermal thickening and hypogranulosis of reconstituted three-dimensional human epidermis models and IL-22-transgenic mice [88]. Apart from the direct effects of IL-22, this mediator also acts via the induction of IL-20 in keratinocytes, which, by partially using the same receptor (IL-22R1), can exert IL-22-like effects [131]. It should be noted that the activity of IL-22 is regulated by IL-22 binding protein [132]. In psoriasis patients, the expression of this natural inhibitor is downregulated in non-lesional skin, and such downregulation is associated with an increased sensitivity of the skin to the pathogenetic action of IL-22 [133]. Increased levels of the T1 cell cytokine IFN-γ may support the activation of dermal endothelia to allow infiltration of immune cells from the bloodstream into the psoriatic lesion [134]. IFN-γ further induces chemokines attracting T1 cells including CXCR10 [135] and upregulates the expression of MHC molecules on both tissue and antigen-presenting immune cells [134]. The pleiotropic and highly inflammatory cytokine TNF-α induces a wide range of immune cell–attracting chemokines and contributes to endothelial activation, two functions necessary for immune cell infiltration [136]. Many effects of cytokines are enhanced in the presence of TNF-α [121, 122, 125], arguing for a central role of this cytokine in skin inflammation.

Another mediator, which can be produced by T17 cells in psoriasis, is IL-29. IL-29 is able to inhibit the replication of viruses via the induction of AVPs [137] and seems to be responsible for the high resistance of psoriatic epidermis toward viral superinfections [94].

#### Immunopathology-based therapy of psoriasis

The excellent knowledge about the specific cytokine pathways involved in psoriasis pathogenesis has allowed the tremendous success in the development of innovative drugs for the treatment of moderate to severe psoriasis [108]. These include therapeutic antibodies that neutralize IL-17 and IL-23 as well as TNF-α (Table 2). A broad range of further drugs is under development [138, 139].

### Treg cells and melanoma

#### Clinical and histological picture of melanoma

The worldwide incidence of cutaneous melanoma has been increasing annually at a more rapid rate compared with any other type of cancer. In 2012, 232,000 new cases of melanoma and 55,000 related deaths were registered worldwide, ranking 15th among most common cancers [140]. About 90% of melanoma cases are diagnosed as primary tumors without evidence of metastasis, and their 10-year survival is between 75 and 80% [141]. Metastases, which can develop either via the lymphatic or the hematogenous route, are the main cause of death in melanoma patients [142]. Disease is subclassified to estimate prognosis and determine therapeutic interventions. This classification considers TNM criteria together with tumor thickness, ulceration, mitotic figures, and microscopic satellites of the primary tumor.

UV light radiation from sunlight, in particular the UV-B spectrum, is the main environmental risk factor for melanoma skin cancer development [143]. Melanoma in chronically sun-exposed skin usually manifests in older-aged individuals and has a high tumor mutational burden related to UV exposure. The main genetic drivers are mutations in the genes encoding B-Raf proto-oncogene (*BRAF*), neurofibromin 1 (*NF1*), *NRAS*, and others. Melanomas associated with intermittently sun-exposed skin cases arise in younger-aged individuals and are usually associated with the BRAF^V600E^ mutation and a lower mutational load [144, 145]. Up to 90% of melanomas exhibit an aberrant MAPK pathway activation as central step in melanoma development [146]. Furthermore, SNPs in genetic loci that associate with the risk for developing malignant melanoma have also been reported in patients. Examples of such genes are *CDKN2A*, *CDK4*, and others. These genetic findings helped to establish small molecular inhibitors of signaling pathways that promote melanoma.

#### Immunopathophysiology of melanoma

Melanoma is deemed one of the most immunogenic types of cancer. In fact, several melanoma-specific antigens have been identified and large numbers of melanoma-specific antibodies and functional lymphocytes are present in patients with melanoma [147]. Moreover, spontaneous regression of melanoma with simultaneous onset of vitiligo has been reported [148] and metastatic melanoma responds to immune-stimulating agents, such as IFNs and IL-2 as well as the novel immune checkpoint inhibitors blocking cytotoxic T lymphocyte-associated antigen-4 (CTLA-4) and PD-1 [149–151]. The major base for the strong immunogenicity of melanoma is its often very high (UV-driven) tumor mutational burden, which allows for the creation of neoantigens recognizable as “non-self” by host immune cells [152, 153]. Accordingly, strong immune cell infiltration is an established positive prognostic parameter in advanced melanoma [154, 155]. Despite the immunogenicity of melanoma and induction of tumor-specific immune responses [156], current immunotherapies show limited efficacy and are restricted to subpopulations of patients with advanced melanoma. It has been suggested that several negative factors hinder antitumor immune activities. These include (i) immune-suppressive cells like Treg cells and myeloid-derived suppressive cells, (ii) anti-inflammatory cytokines like tumor growth factor (TGF)-β and IL-10, (iii) defective antigen presentation by tumor cells because of antigen expression loss and antigen processing defects, (iv) immune inhibitory molecules like CTLA-4 and PD-1, and (v) amino acid–catabolizing enzymes like arginase and indoleamine-2-3 dioxygenase (IDO) [157].

In both animal models and human beings, Treg cells infiltrate into the tumor microenvironment, dampening immune responses to tumor cells [158–160]. Cell-to-cell contact, production of immune-suppressive cytokines like IL-10 and TGF-ß, competing for growth factors with other effector cells, and modification of APCs are the four main strategies how Treg cells apply to exert their inhibitory effects [161, 162]. As most tumor antigens are normal self-antigens, such tumors could induce tumor-specific Treg cells, suppressing effective antitumor responses [163, 164]. In animal models of melanoma, transient Treg cell depletion induces immune responses against tumor and improves survival, indicating the importance of these cells [165]. Wang et al. were the first to isolate Treg cells that recognize epitopes from the tumor-associated antigen LAGE-1 from patients with melanoma, providing evidence for the relevance of this mechanism also in the melanoma setting [166]. Tumor-specific Treg cells that can recognize a broad range of melanoma-associated antigens and neoantigens can be detected in the tumors and in the blood of melanoma patients [167, 168]. Fourcade et al. showed that the same melanoma-associated antigens can stimulate both Th and Treg cells [169]. As a consequence, immunotherapeutic vaccinations with melanoma-associated antigens in patients with melanoma can result in expansion of both induced and naturally occurring melanoma-associated Treg cells [170].

Numerous researches have indicated increased numbers of Treg cells not only in the local tumor microenvironment including primary and metastatic lesions but also in peripheral blood of subjects with metastatic melanoma, as well as in affected draining lymph nodes [159, 160]. Treg cell accumulation in the tumor microenvironment was reported to be predictive of reduced survival of melanoma patients [171]. Subsequently, several other retrospective studies demonstrated the correlation between Treg cell infiltration and prognosis of melanoma patients [172]. Vice versa, the parameter that best correlates with favorable clinical outcome and survival of melanoma patients seems to be the ratio of CD8-positive effector T cells to Treg cells in the tumor microenvironment [173]. The chemokine CCL22 is known to mediate CCR4^high^ Treg cell trafficking into tumors [174]. The CCR4-mediated Treg cell attraction into melanomas, however, seems to be caused by the alternative CCR4 ligand CCL2 [175].

**Fig. 5:**
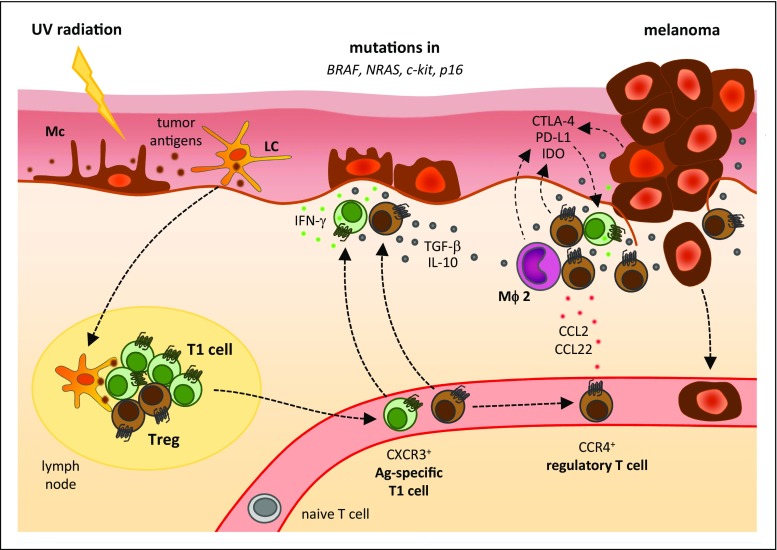
Immunopathophysiology of melanoma

Immunosuppressive factors that are locally secreted by melanomas, such as TGF-ß and IL-10, could promote both expansion of naturally occurring Treg cells and de novo generation of induced Treg cells [165]. Likewise, molecular mechanisms of tumor immunosuppression mediated by IDO have a direct anergic effect on effector T cells and enhance local Treg cell–mediated immunosuppression. Moreover, expression of IDO on tumor-infiltrating APCs stimulates the conversion of conventional T cells to Treg cells [176]. Upregulation of IDO expression in melanoma lymph-node metastases is associated with an increased number of tumor-infiltrating Treg cells and consequently shorter patient survival [177]. Interestingly, very recently, it has been reported in an inducible autochthonous model of melanoma that the expression of the oncogenic BRAF^V600E^ mutation in melanocytes resulted in nevus formation, CCR4 induction, and Treg cell recruitment [178]. This suggests the BRAF^V600E^ signaling is sufficient to recruit the Treg cells to melanomas and might add an additional mechanism for explaining the therapeutic activity of BRAF inhibition in patients with metastatic melanoma (see below). The pathophysiology of melanoma is depicted in Fig. 5.

#### Immunopathology-based therapy of melanoma

As mentioned above, the majority of patients with newly diagnosed melanoma have early-stage disease, for which surgical excision represents the treatment of choice and is curative in the majority of cases [179]. However, approximately 10% of melanoma cases are diagnosed at an advanced stage and are unresectable or already metastatic. Due to the known immunogenicity of melanoma, experimental immunotherapy had a prominent position in the treatment of melanoma for decades.

Collected data from several clinical trials evaluating the efficacy of recombinant IL-2 therapy in the 1980s showed that a small fraction of melanoma patients experienced durable complete responses. Based on these results, in 1998, the FDA approved IL-2 for the treatment of unresectable melanoma [180] (Table 2). IL-2 is a key regulator in supporting proliferation and homeostasis of effector T cells but is also crucial for the development of Treg cells, therefore simultaneously leading to increased numbers of Treg cells in melanoma patients [167, 181]. However, IL-2 was also described to mask the suppressive function of Treg cells on effector T cell proliferation [182].

Since Treg cell–mediated immunosuppression is generally deemed one of the main hurdles for cancer immunotherapy, various approaches for depletion and/or modulation of Treg cells (cyclophosphamide, denileukin diftitox, anti-CD25 antibody (daclizumab), anti-CD25 immunotoxin) have been characterized and tested with different clinical outcome. New such experimental approaches include an anti-CCR4 antibody for Treg cell depletion as well as an agonistic antibody against the glucocorticoid-induced tumor necrosis factor receptor (GITR) for modulation of Treg cell activity [159, 160, 165].

The main treatment for melanoma patients in the early stages is surgical resection. For a long period, the only treatments for patients with metastatic melanoma included chemotherapy with dacarbazine and some other agents as well as immunotherapy with high doses of IL-2. In the last 10 years, MAP kinase pathway–targeted therapies (BRAF and MEK inhibitors) and immune checkpoint inhibitors blocking CTLA-4 and PD-1 have revolutionized the management of advanced melanoma and significantly prolonged patient survival [149, 151] (Table 2).

In BRAF^V600E^-mutated melanoma, the combination of BRAF and MEK inhibitors has led to high response rates (70%) and rapid response induction and symptom control, with a significant prolongation of progression-free survival [183, 184]. Interestingly, it was reported that BRAF inhibition could promote the immune response to melanoma [185].

CTLA-4 is an inhibitory receptor that is constitutively expressed by Treg cells. CTLA-4 binds to CD80 and CD86 on APCs and acts as a key negative regulator of peripheral T cell proliferation and function. In mice models, treatment with monoclonal anti-CTLA-4 antibodies increased the local infiltration of cytotoxic T cells while dramatically reducing Tregs at the tumor site [173, 186]. The human anti-CTLA-4 antibody ipilimumab was demonstrated to be effective in a phase III clinical trial and received FDA approval for treating metastatic melanoma in 2011 [187]. Remarkably, meta-analyses demonstrated the durability of long-term survival in a significant number of ipilimumab-treated patients [188]. Whether there is a depletion or reduction of Treg cells in melanoma microenvironment as mechanism of therapeutic anti-CTLA-4 antibodies in the patients is still under debate [189–192]. The PD-1 receptor is expressed on Treg cells and activated effector lymphocytes. It binds to PD-L1 and PD-L2, acts as a T cell co-inhibitory molecule, and suppresses T cell activation. Nivolumab is a high-affinity anti-PD-1 monoclonal antibody that inhibits the binding between the PD-1 receptor and its ligands PD-L1 and PD-L2. Nivolumab was approved (2014) by the FDA for the treatment of patients with metastatic melanoma [193]. Pembrolizumab, another anti-PD-1 antibody, was approved by the FDA in 2015 for the treatment of advanced melanomas [184]. Interestingly, a very recent paper suggests that PD-1 blockade can decrease the suppressive function of Tregs in vitro and that the therapeutic benefit of nivolumab in melanoma patients corresponds to a decreased suppressive function of blood Treg cells [194]. Moreover, a quite current paper described significantly increased responses to anti-PD1 in aged melanoma patients and correlated this with higher CD8+ effector /FoxP3+ Treg cell ratios in the tumor microenvironment in this population [195]. Interestingly, in some patients with successful anti-tumor response by checkpoint inhibitors, vitiligo-like depigmentation can be observed.

## Conclusion

Chronic T cell–mediated skin diseases represent a major health economy problem worldwide. Pathogenetically, these diseases are based on different mechanisms that are closely related to effector functions of respective T cell subtypes. Activity of T1 cells leads to destruction of cells expressing antigens recognized by these T cells. For example, when respective antigens are carried by melanocytes, the stimulation of the antigen-specific T1 cells results in pigment-free skin patches that characterize vitiligo. The activation of T2 cells leads to IL-4, IL-5, and IL-13 secretion, provoking skin barrier alteration, immune cell infiltration into skin, and itch as observed in atopic dermatitis. The stimulation of T17 and T22 cells is associated with highly increased production of IL-17, IL-22, and TNF-α that promote the proliferation of keratinocytes and alter their terminal differentiation. These cellular changes result in sharply demarcated, reddish-colored raised plaques with superficial silvery scaling as observed in psoriasis. In many other immune-mediated skin diseases, the exact phenotype of disease-responsible T cells is less clear. Examples are diseases such as lichen planus, hidradenitis suppurativa, or pemphigus. Apart from the dysregulated activity of T1, T2, T17, and T22 effector T cells, an increased activity of Treg cells can be involved in the development of skin diseases. An impressive example for this is skin melanoma, a malignant tumor of melanocytic origin, which presents as hyper-pigmented maculae or nodules. In that disease, local Treg cell–mediated immunosuppression is thought to be responsible for the dampened immune response (mainly T1) to the tumor cells.

The understanding of the immunopathogenetic mechanisms involved in skin diseases opens up great opportunities for the development of targeted therapeutic approaches for the respective patients. In fact, while, on the one hand, our knowledge of T cell biology has allowed the development of efficient strategies to control, e.g., psoriasis, the great success of these strategies in dermatology has, vice versa, decidedly contributed to the understanding of T cell biology and the pathways they are involved in. Further studies in immunodermatology are needed to improve the treatment options for many other inflammatory and neoplastic skin diseases beyond vitiligo, psoriasis, atopic dermatitis, and melanoma.
